# Preclinical *In Silico* Evidence Indicates the Pharmacological Targets and Mechanisms of Mogroside V in Patients With Ovarian Cancer and Coronavirus Disease 2019

**DOI:** 10.3389/fendo.2022.845404

**Published:** 2022-04-06

**Authors:** Yongming Li, Yudong Chen, Mulan Wei, Chaohe Wei

**Affiliations:** ^1^ Department of Gynecology, Guigang Maternal and Child Health Care Hospital, Guigang, China; ^2^ Department of Gynecology, Guigang City People’s Hospital, The Eighth Affiliated Hospital of Guangxi Medical University, Guigang, China; ^3^ Department of Pharmacy, Guigang City People’s Hospital, the Eighth Affiliated Hospital of Guangxi Medical University, Guigang, China

**Keywords:** COVID-19, ovarian cancer, mogroside V, preclinical report, remedial targets

## Abstract

The borderless transmission of coronavirus remains uncontrolled globally. The uncharted severe acute respiratory syndrome coronavirus 2 (SARS-CoV-2) variant reduces the therapeutic efficacy of vaccines against coronavirus disease 2019 (COVID-19). Clinical observations suggest that tumour cases are highly infected with coronavirus, possibly due to immunologic injury, causing a higher COVID-19-related death toll. Presently, screening of candidate medication against coronavirus is in progress. Mogroside V, a bioactive ingredient of *Siraitia grosvenorii*, has been reported in China to have lung-protective and anticancer effects. The current study used network pharmacology and molecular docking to unlock the potential drug targets and remedial mechanisms of mogroside V against patients with ovarian cancer with COVID-19. We identified 24 related targets of mogroside V in patients with ovarian cancer and COVID-19 and characterised another 10 core targets of mogroside V against COVID-19 ovarian cancer, including Jun, IL2, HSP90AA1, AR, PRKCB, VEGFA, TLR9, TLR7, STAT3, and PRKCA. The core targets’ biological processes and signalling pathways were revealed by enrichment analysis. Molecular docking suggested favourable docking between core target protein and mogroside V, including vascular endothelial growth factor A (VEGFA). These findings indicated that mogroside V might be a potential therapeutic agent in the mitigation of COVID-19 ovarian cancer.

## Introduction

Coronavirus disease 2019 (COVID-19) is caused by the severe acute respiratory syndrome coronavirus 2 (SARS-CoV-2), and this coronavirus is characterised by fast transmission worldwide and virulence (1). Given the mutations, the persistent infection in the population has been unprecedented; the lack of vaccination is another cause (2), such that massive death tolls have been reported on a real-time basis by the World Health Organization (https://worldhealthorg.shinyapps.io/covid/). Some COVID-19 cases are accompanied by mortiferous symptoms, including acute respiratory distress (3). It has been observed that infection in patients with cancer with SARS-CoV-2 is more severe than in healthy patients (4). Angiotensin-converting enzyme 2 (ACE2) may be a key receptor for SARS-CoV-2 infection.

Additionally, ACE2 is overexpressed in the interferon-gamma (IFN-γ) immune subtype of ovarian serous cystadenocarcinoma, indicating an increased risk of infection with SARS-CoV-2 (5). Epithelial ovarian neoplasm is one of most common causes of gynaecological tumour-related death (6). The onset of ovarian cancer can be asymptomatic but may gradually develop from hyperplasia to mucinous adenocarcinoma (7). A majority of ovarian cancers are diagnosed in their late stage, characterised by high invasion and poor prognosis (8). As reflected in molecular pathogenesis, some key gene mutations, including those of *BRCA*, *RAD51B*, *PTEN*, *CCNE1*, and tumour-infiltrating lymphocytes, may promote ovarian cancer progression (9). A prospective study from the United States has reported that patients with ovarian cancer may experience more anxiety and depression during the COVID-19 pandemic, especially BRCA1/2-positive women (10).

Additionally, it has been concluded that malignant cancer mortality may be higher in patients with COVID-19, especially in case of a severe infection (11). Therefore, we propose that identifying suitable pharmacological targets is required to prevent COVID-19 infections in patients with ovarian cancer. Traditional Chinese medicine (TCM) has been used as an adjunctive treatment for COVID-19 in China, including Lianhua Qingwen Keli and Xuebijing injections (12). In addition, TCM may be effective in treating complications of COVID-19, including severe cases (13).

Mogroside V, a triterpenoid glycoside isolated from Siraitia grosvenorii, has wide-ranging pharmacological activities, such as preventing asthma or tussis and reducing phlegm (14). In addition, mogroside V has been shown to exert anticancer effects *in vivo* (15) and overt inhibitory effects on lung cancer *in vitro* (16). However, the potential anti-ovarian cancer efficacy of mogroside V and its molecular mechanisms are unreported. Therefore, network pharmacology-based strategies may unveil, *in silico*, essential data on TCM active ingredients combating diseases, core targets, and therapeutic mechanisms (17). Our previous study revealed plumbagin’s therapeutic targets and molecular mechanisms against uterine corpus endometrial carcinoma and COVID-19 (18). Therefore, our study aimed at identifying possible core targets and pharmacological mechanisms of mogroside V treating ovarian cancer and COVID-19 to aid experimental and clinical validation.

## Materials and Methods

### Acquisition of Targets of Mogroside V in COVID-19 and Ovarian Cancer

Mogroside V-linked targets were identified using the Swiss Target Prediction, PharmMapper, and Batman databases. Targets of drug action were screened after review (Swiss-Prot) for target correction using the UniProt database (19). GeneCards, DrugBank, Online Mendelian Inheritance in Man (OMIM) (20), and therapeutic target databases searched for COVID-19 and ovarian cancer-related targets. Target intersections of COVID-19 with ovarian cancer and mogroside V were mapped using the bioinformatics online tool Venn diagram to identify the intersection targets between COVID-19 and ovarian cancer before producing other intersection targets between mogroside V and COVID-19/ovarian cancer.

### Mogroside V Anti-COVID-19/Ovarian Cancer Core Target Screening and Network Construction

Mogroside V anti-COVID-19/ovarian cancer action targets were obtained from the STRING (21) database to establish target–target function-related protein interactions with a minimum reciprocal value of 0.09. The results were imported into Cytoscape_v3.6.1 (22) to construct the mogroside V anti-COVID-19/ovarian cancer target-related protein–protein interaction (PPI) network. NetworkAnalyzer in Cytoscape_v3.6.1 was used to calculate topological parameters, such as median degrees of freedom and maximum degrees of freedom in networks. The core targets were screened according to degree values. The upper limit of the filtering range was the maximum degree value in the topological data, with the lower limit being the median degrees of freedom.

### Gene Ontology and KEGG Pathway Enrichment Analysis of Core Targets

The DAVID database was used to obtain the interactions between the core targets and related biological processes (BPs) and the Kyoto Encyclopedia of Genes and Genomes (KEGG) pathways. R packages such as “GOplot” in R language (3.6.1) were used to visualise the Gene Ontology (GO) and KEGG enrichment data related to core targets, and output data were created for the corresponding bubble chart and histogram. The data were then processed and imported into an online bioinformatics tool to obtain a Circos circle map.

### Graphical Construction of Network Relationship Visualization

Drug–target–gene ontology function–pathway–disease graphs were created using Cytoscape_v3.7.1 for mogroside V anti-COVID-19/ovarian cancer using biological processes and pathway enrichment results.

### Molecular Docking Validation

The core target with the largest degree value was used for molecular docking validation. The structure of the mogroside V compound was obtained from the PubChem database (https://pubchem.ncbi.nlm.nih.gov/). The corresponding protein structure was obtained from the Protein Data Bank (PDB) database (https://www.rcsb.org/). The 3-dimensional structure details of mogroside V were downloaded using the ChemBio3D Draw module of the ChemBioOffice 2010 software for optimising the MM2 force fields. The raw format was converted to pdbqt format recognised by the AutoDock Vina program using raccoon software. By using AutoDock Vina software corresponding to the protein, hydrogen addition, Gasteiger charge, and merging of non-polar hydrogen were saved in the pdbqt file format. The active centre of docking (containing all residues around the original ligand as the centre) was set using the grid box function in the software. The reasonableness of the docking parameter setting was judged according to the magnitude of the root mean square deviation (RMSD) of the docked ligand molecule from the original ligand molecule. An RMSD of less than 4 Å was considered as the threshold value for the conformational match of the docked ligand with the conformation of the initial ligand (23).

## Results

### Identification of Mogroside V and COVID-19/Ovarian Cancer Targets

**Figure 1:**
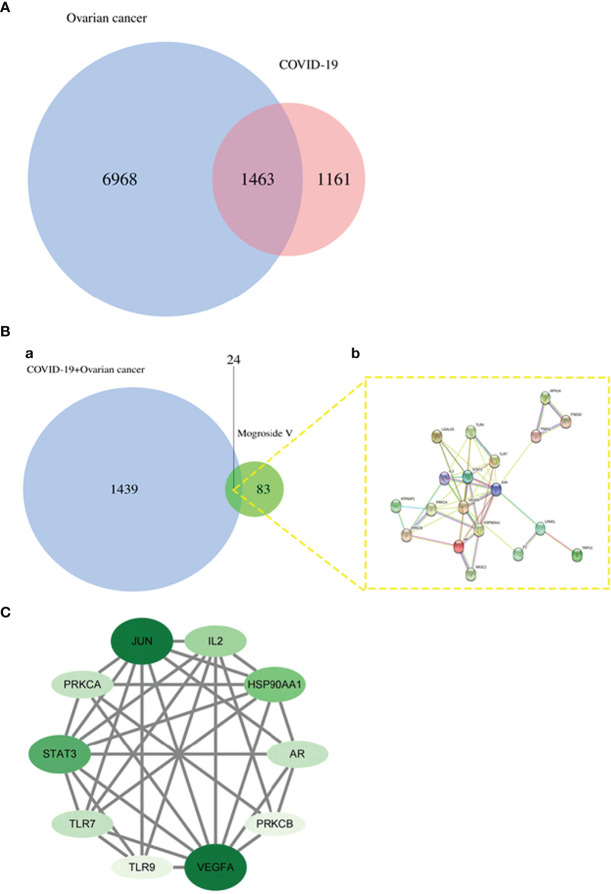
Bioinformatics and network pharmacology analyses. **(A)** Ascertainment of 1,463 COVID-19 and ovarian cancer-related genes. **(B)** The Venn diagram identifies 24 common genes **(a)** in mogroside V and COVID-19/ovarian cancer, followed by an associated diagram within these common genes **(b)**. **(C)** Screening of 10 core targets in mogroside V against COVID-19/ovarian cancer.

A total of 2,674 COVID-19 genes and 8,710 ovarian cancer genes were identified. A total of 107 mogroside V targets were obtained using UniProt database correction after removing duplicate items. A total of 1,463 COVID-19 and ovarian cancer intersection targets were identified (**Figure 1A**). Other 24 intersection targets in mogroside V and COVID-19/ovarian cancer were obtained by processing through an online bioinformatics tool. The function-related protein interaction network was constructed accordingly (**Figure 1B**).

### PPI and Topological Parameter Analysis Data of Core Targets

The mapped intersection proteins were introduced into the Cytoscape tool, and the topological parameters of mogroside V acting on the protein interaction network associated with COVID-19/ovarian cancer targets were calculated. The median degree of freedom of the COVID-19/ovarian cancer target network of mogroside V was 5, the maximum freedom was 11, and the core target filter range was 5–11. As a result, 10 core targets were obtained, namely, JUN, IL2, HSP90AA1, AR, PRKCB, VEGFA, TLR9, TLR7, STAT3, and PRKCA (**Figure 1C**).

### Gene Ontology and KEGG Pathway Enrichment Analysis

**Figure 2:**
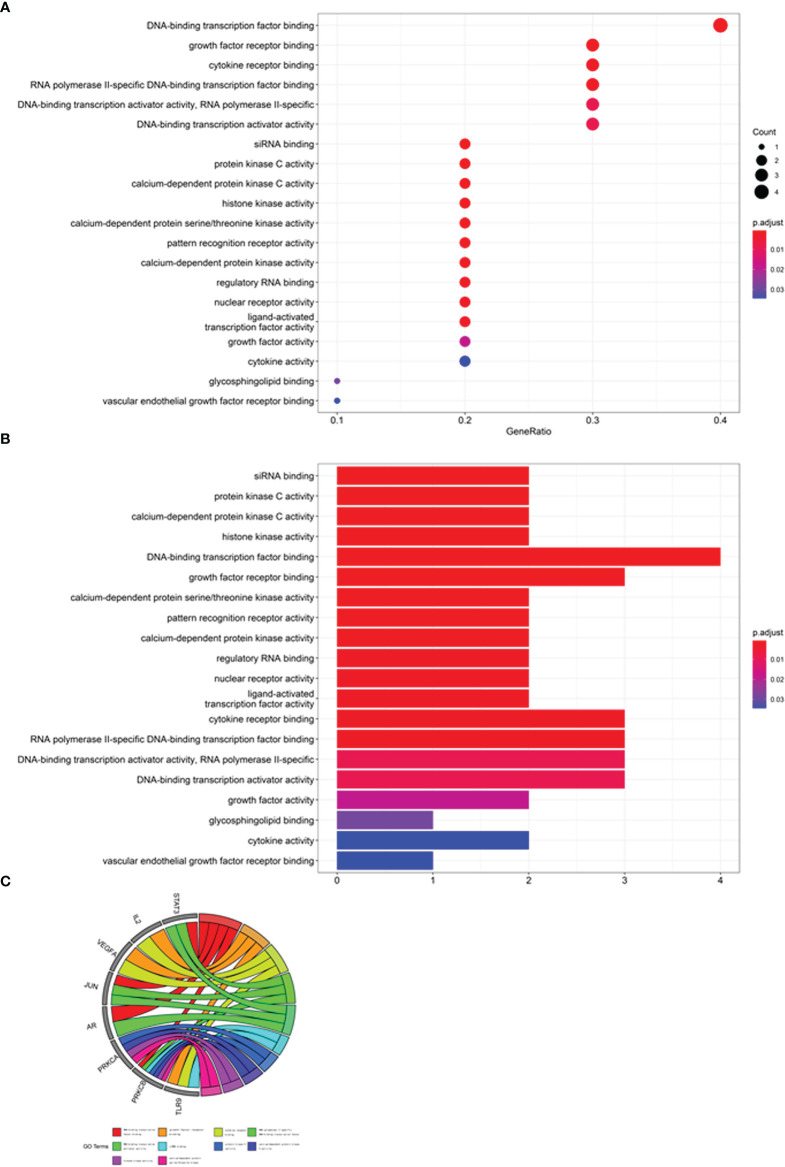
GO enrichment analysis of core targets. Biological functions involved in GO characterised in bubble chart **(A)**, bar chart **(B)**, and circle diagram **(C)**.

**Figure 3:**
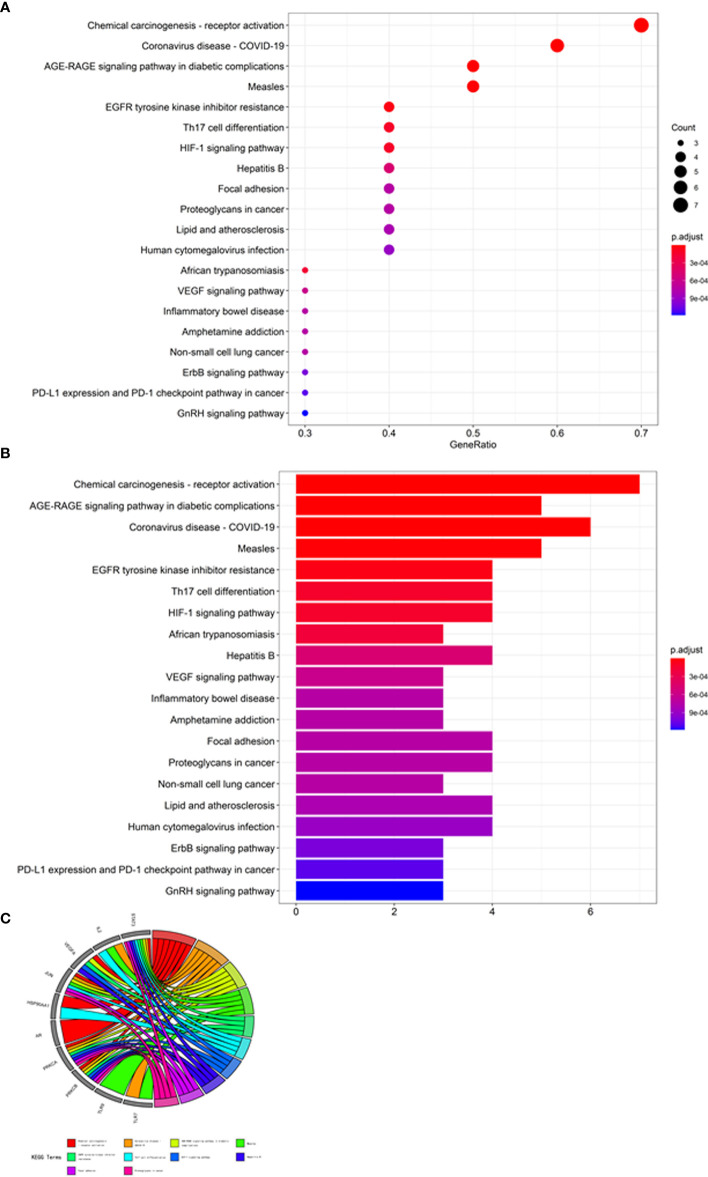
KEGG enrichment analysis of core targets. Molecular mechanisms associated with KEGG signalling pathways are revealed in bubble chart **(A)**, bar chart **(B)**, and circle diagram **(C)**.

The core target-related enrichment data for mogroside V against COVID-19/ovarian cancer were output in the bubble chart (**Figure 2A**), histograms (**Figure 2B**), and Circos circles (**Figure 2C**). In addition, other bubble diagrams (**Figure 3A**), histograms (**Figure 3B**), and Circos circles (**Figure 3C**) in the KEGG pathway enrichment were characterised. The results showed that the relevant BPs on mogroside V action against the COVID-19/ovarian cancer target mainly involved binding of DNA-binding transcription factor, growth factor receptor, cytokine receptor, RNA polymerase II-specific DNA-binding transcription factor and siRNA, and activities of DNA-binding transcription activator, protein kinase C, calcium-dependent protein kinase C, and histone kinase. The core targets related to cellular components (CCs) were chiefly associated with the endoplasmic reticulum, nucleus, protein complex, cytosol, caveola, cytoplasm, and nucleoplasm. The KEGG pathway associated with core targets mostly involved chemical carcinogenesis-receptor, coronavirus disease-COVID-19, and AGE-RAGE signalling pathway in diabetic complications, measles, EGFR tyrosine kinase inhibitor resistance, Th17 cell differentiation, HIF-1 signalling pathway, hepatitis B, focal adhesion, proteoglycans in cancer, lipid and atherosclerosis, and human cytomegalovirus infection.

### Construction of Network Diagrams

**Figure 4:**
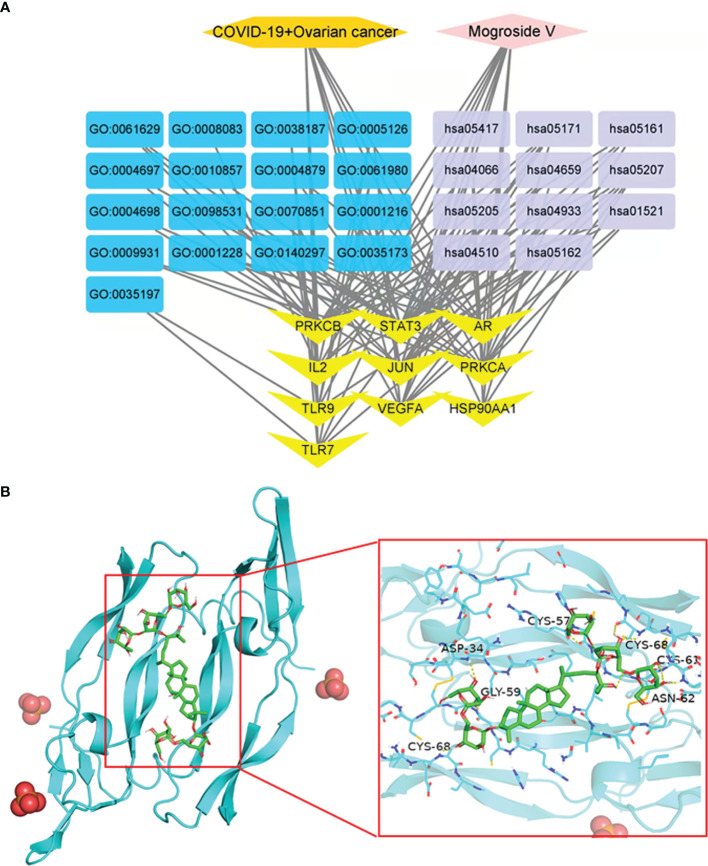
Network diagram of mogroside V in COVID-19/ovarian cancer **(A)**. Molecular docking analysis of mogroside V with VEGFA. Mogroside V formed hydrogen bonds with CYS-68, ASP-34, CLY-59, CYS-57, CYS-68, CYS-61, and ASN-62 residues **(B)**.

Drug–target–BP–KEGG–disease visualisation graphs were constructed to characterise the network diagrams of core gene-related biological processes and pathway enrichment findings of mogroside V against COVID-19/ovarian cancer (**Figure 4A**).

### Molecular Docking Data

The maximum degree value of the VEGFA core target protein was determined, the relevant protein was molecularly docked, and an appropriate active cavity box model was established. In VEGFA (PDB ID: 6ZFL), the free binding energy of mogroside V to VEGFA protein was -8.2 kcal/mol, characterised by hydrogen bonding with amino acid residues in CYS-68 (2.7 Å), ASP-34 (2.7 Å), CLY-59 (2.9 Å), CYS-57 (3.5 Å), CYS-68 (2.4 Å), CYS-61 (2.7 Å), and ASN-62 (2.4 Å) (**Figure 4B**).

## Discussion

Based on network pharmacology analysis, the current study identified 24 candidate targets and 10 core targets for mogroside V for treating ovarian cancer and COVID-19. In addition, enrichment analysis has revealed the therapeutic functions and signalling pathways in mogroside V treatment of ovarian cancer and COVID-19, such as cytokine receptor binding and cytokine activity in biological processes, HIF-1 signalling pathway, and Th17 cell differentiation in molecular mechanisms.

In clinical observation, cytokine profiling in patients with a severity spectrum of COVID-19 has been characterised (24). Cytokine storm is related to an increased death rate in patients with COVID-19, and the suppression of cytokine-induced injury may be the screening parameter of effective COVID-19 drugs (25). The development of SARS-CoV-2 is dependent on the cellular microenvironment, and modulation of HIF signalling may affect multiple functions during SARS-CoV-2 infection (26). Endogenous HIF-1α activity plays a key role in facilitating SARS-CoV-2 infection, causing aggravation of inflammatory lesions in patients with COVID-19 (27). It is theorised that inhibition of HIF-1α activity or activation of the HIF-1 signalling pathway may be used to prevent or treat COVID-19. The vital role of immune cells, including that of Th17 cells, against SARS-CoV-2 infections is clinically evident through the regulation of immune responses (28). However, other studies have indicated that Th17 cells can function in the pathogenesis of COVID-19 by inducing cytokine release and Th2 responses, thereby restraining Th1 differentiation and Treg cell proliferation (29). The Th17-related pro-inflammatory response may be a part of SARS-CoV-2 infection immunopathology, resulting in the development of systemic hyperinflammation (30). Thus, the reduction of excessive inflammation through the modulation of the Th17 cell differentiation signalling pathway may be an immunomodulatory strategy to relieve COVID-19.

Further analysis using molecular docking suggested potent binding scores between mogroside V and the core target protein in ovarian cancer and COVID-19. VEGFA, a core protein docked well with mogroside V, has been identified as a pharmacological target in mogroside V treatment of ovarian cancer and COVID-19. VEGFA has been widely proven to be a functional pro-angiogenic cytokine in vertebrate development and vascular homeostasis (31). A prospective case–control study showed that VEGFA in women with COVID-19 was associated with some adverse outcomes (32). Clinically, VEGF may induce vascular impairment in gastrointestinal cells infected with SARS-CoV-2 (33). Thymus stromal lymphopoietin derived from tumour cells can increase the expression of VEGFA, thereby inducing angiogenesis (34). Neutrophils play an important role in promoting tumour angiogenesis by producing pro-angiogenic factors such as VEGFA (35). Bevacizumab, a monoclonal antibody targeting VEGFA, can inhibit vascular endothelial growth factors and can be used to treat various metastatic cancers (36). VEGFA is a potential agent to treat ovarian tumours (37). In addition, suppression of VEGFA activation may enhance the therapeutic effectiveness of ovarian tumour chemotherapy through inhibition of autophagy related to VEGFA (38). It has been shown preclinically that targeting VEGFA activity or its signalling pathway is one of the pharmacological mechanisms of action of traditional Chinese medicines in cancers (39, 40). However, the biological mechanisms of anti-ovarian cancer effects of mogroside V associated with VEGFA remain unreported. Based on current bioinformatics analyses, VEGFA core target proteins may play a key role in preventing and treating ovarian cancer with COVID-19. Interestingly, our bioinformatics findings identified drug targets and pharmacological mechanisms of mogroside V for treating COVID-19 ovarian cancer. Although bioinformatics data reported a correlation with the therapy of COVID-19 ovarian cancer, there are still some limitations in the current study. To begin with, experimental validation is absent in current bioinformatics findings. The docking capabilities *in silico* between mogroside V and core proteins are then needed to be validated experimentally. Therefore, we still need to verify the current data through further *in vitro* and *in vivo* experiments.

## Conclusion

Our preclinical findings identified core targets and pharmacological mechanisms of mogroside V in treating ovarian cancer and COVID-19. Mogroside V may treat COVID-19 ovarian cancer by targeting JUN, IL2, HSP90AA1, AR, PRKCB, VEGFA, TLR9, TLR7, STAT3, and PRKCA. However, all these bioinformatic findings need to be verified before clinical application.

## Data Availability Statement

The original contributions presented in the study are included in the article/**Supplementary Material**. Further inquiries can be directed to the corresponding author.

## Author Contributions

CW, YL: conceptualization. YC, MW: data curation. CW, YL: investigation. YL, YC, MW: methodology. YL, YC, MW: software. CW: supervision. CW, YL: roles/writing—original draft. All authors contributed to the article and approved the submitted version.

## Conflict of Interest

The authors declare that the research was conducted in the absence of any commercial or financial relationships that could be construed as a potential conflict of interest.

## Publisher’s Note

All claims expressed in this article are solely those of the authors and do not necessarily represent those of their affiliated organizations, or those of the publisher, the editors and the reviewers. Any product that may be evaluated in this article, or claim that may be made by its manufacturer, is not guaranteed or endorsed by the publisher.
